# International subarachnoid aneurysm trial – ISAT Part II: Study protocol for a randomized controlled trial

**DOI:** 10.1186/1745-6215-14-156

**Published:** 2013-05-29

**Authors:** Tim E Darsaut, Andrew S Jack, Richard S Kerr, Jean Raymond

**Affiliations:** 1 University of Alberta, Department of Surgery, Division of Neurosurgery, 2D.1 Mackenzie Health Sciences Center, 8440 – 112 St, Edmonton, AB T6G 2B7, Canada; 2 Neurovascular Research Unit, Nuffield Department of Surgery, University of Oxford and Oxford Radcliffe Hospitals NHS Trust, John Radcliffe Hospital, Headley Way, Headington, Oxford, Oxfordshire OX3 9DU, UK; 3 Department of Radiology, Centre Hospitalier de l’Universite de Montreal, Notre-Dame Hospital, 1560 Sherbrooke East, Pavillion Simard, Room Z12909, Montreal, QC H2L 4M1, Canada

**Keywords:** Endovascular, Intracranial aneurysm, Randomized trial, Ruptured aneurysm, Surgery, Trial protocol

## Abstract

**Background:**

The International Subarachnoid Aneurysm Trial (ISAT) demonstrated improved one-year clinical outcomes for patients with ruptured intracranial aneurysms treated with endovascular coiling compared to surgical clipping. Patients included in ISAT were mostly good grade subarachnoid hemorrhage (SAH) patients with small anterior circulation aneurysms. The purported superiority of coiling is commonly extrapolated to patients not studied in the original trial or to those treated using new devices not available at the time. Conversely, many patients are treated by clipping despite ISAT, because they are thought either to be better candidates for surgery, or to offer more durable protection from aneurysm recurrences. These practices have never been formally validated. Thus, for many ruptured aneurysm patients the question of which treatment modality leads to a superior clinical outcome remains unclear.

**Methods/trial design:**

ISAT II is a pragmatic, multicenter, randomized trial comparing clinical outcomes for non-ISAT patients with subarachnoid hemorrhage allocated to coiling or clipping. Inclusion criteria are broad. The primary end-point is the incidence of poor clinical outcome (defined as mRS >2) at one year, just as in ISAT. Secondary end-points include measures of treatment safety for a number of pre-specified subgroups, with efficacy end-points including the presence of a major recurrence at one year; 1,896 patients (862 each arm plus 10% losses) are required to demonstrate a significant difference between coiling and clipping, hypothesizing 23% and 30% poor clinical outcome rates, for coiling and clipping, respectively. The trial should involve at least 50 international centers, and will take approximately 12 years to complete. Analysis will be by intention-to-treat.

**Trial registration:**

ISAT II is registered at clinicaltrials.gov: NCT01668563.

## Background

To prevent re-bleeding from ruptured intracranial aneurysms, subarachnoid hemorrhage (SAH) patients are often treated by surgical clipping [1,2] or, in the last 20 years, endovascular coiling [3]. Very little randomized evidence has guided clinical decision making in neurovascular surgery since the early trials pioneered by McKissock [4] until the publication of the International Subarachnoid Aneurysm Trial (ISAT) in 2002. ISAT was a turning point in modern neurosurgical history [5]. The trial showed that for 2,143 SAH patients eligible for both surgery and endovascular coiling recruited between 1994 and 2002, randomized allocation to coiling was associated with better one-year clinical outcomes defined as survival without dependency (absolute risk reduction (ARR) of 7.4% (95% CI: 3.6 to 11.2, *P* = 0.0001). Such a ground-breaking result was bound to be received with some resistance, expressed in multiple editorials and commentaries (reviewed in [6]). However, because ISAT was a positive pragmatic trial, an appropriate interpretation of the results was that coiling should be adopted as the first-line treatment for ruptured aneurysms, at least for patients with the types of lesions included in ISAT. These were, for the great majority, small (≤10 mm) anterior circulation aneurysms [6]. Although ISAT was well designed, conducted, and reported, a single trial cannot answer all important clinical questions, and trial results were perhaps inappropriately extrapolated beyond what ISAT demonstrated [7]. Coiling has progressively replaced clipping in many centers, even for many patients that would not have been studied in ISAT [7]. Unfortunately, there is no current evidence to support coiling in the wide spectrum of non-ISAT patients and aneurysms that are currently treated endovascularly.

A recent pre-randomized study of coiling as first-intention confirmed better results for those ruptured aneurysms felt to be readily coilable; however only 62% of patients allocated to coiling were actually coiled, 38% were crossed-over to be clipped [8]. Ten years following the publication of ISAT, the optimal management of more difficult-to-coil ruptured aneurysms remains unclear.

Multiple new devices have been introduced during the last decade. This has expanded the spectrum of cases that can be treated using endovascular techniques, though no evidence is available to confirm that these patients should not be clipped instead.

Angiographic recurrences (occurring in 10% to 33% of patients [9-11]) has raised concerns that aneurysm coiling may not be as durable in the long-term as surgical clipping. ISAT patients allocated to coiling were more frequently retreated (17%) than those allocated to clipping (4%). However, clipped patients were not as rigorously followed angiographically [12,13]. Retreatments were much less frequent in the more recent Cerecyte (5.5%) and HELPS trials (3%) [10,11]. Offering a more durable protection from late re-bleeding is still a frequent reason why many neurosurgeons continue to clip ruptured aneurysms in spite of ISAT results. Furthermore, publication of the 5-year follow-up data showed that the risk of death at 5 years was significantly lower with coiling (11% versus 14%; *P* = 0.03) but the proportion of survivors that were independent did not differ between the two groups [12].

Although coiling is increasingly used in most countries, clinical practices vary tremendously, with some centers coiling as low as 20% of ruptured aneurysms, with other centers coiling upwards of 70% of ruptured aneurysms [7,14-16], depending on training, local expertise, and preferences, as well as biased data from non-randomized case series and registries [17]. Thus, for many patients the uncertainty regarding the best choice of treatment modality persists and another randomized trial is needed to offer optimal care in the presence of such uncertainty.

## Methods: Trial design

ISAT II is a randomized, multicenter, pragmatic trial comparing clinical outcomes for patients with ruptured intracranial aneurysms treated with endovascular treatment or with surgical clipping. All patients with ruptured aneurysms who are still being considered for surgical clipping in spite of ISAT results, and all those with ruptured aneurysms which were not well-represented in the original ISAT study or for which the use of new devices such as stents is contemplated, will be proposed participation in the trial. The study will be conducted in approximately 50 centers performing both surgical clipping and endovascular coiling of aneurysms, after approval from local ethics committees and with informed consent from participating patients, aiming to enroll at least 10 patients per year per center, and thus requiring approximately 10 years to recruit 1,896 patients. ISAT II will follow the principles enunciated in the Declaration of Helsinki. The complete protocol is available at http://www.clinical-care-trials.org.

### Primary hypothesis of ISAT II

Endovascular management of patients with ruptured, intradural, intracranial aneurysms suitable for both endovascular and surgical management, for whom the treating physician remains unsure whether the results of the original ISAT study apply, will lead to a decrease in the number of poor outcomes (defined as modified ranking scale (mRS) >2) from 30% to 23% at one year.

### Study endpoints

The primary end-point, as for the original ISAT study, will be the number of patients experiencing a poor clinical outcome (mRS >2) at one year post-treatment. Secondary end-points include overall mortality at one year (all causes), overall morbidity at one year (all causes), occurrence of a major (saccular) aneurysm recurrence at one year post-treatment, peri-treatment hospitalization lasting more than 15 days, discharge to a location other than home, occurrence of an intracranial hemorrhage after enrollment and for up to one year, occurrence of aneurysm re-rupture following randomization but before treatment initiation, and occurrence of failure of aneurysm occlusion using the intended treatment modality.

### Planned trial interventions

Surgical clipping or endovascular coiling is performed once for each patient. In the setting of multiple aneurysms, more than one aneurysm can be treated in the same sitting. Treatment will be initiated as soon as possible given local logistical constraints, according to standard of care of patients with ruptured aneurysms. Aneurysms thought by the treating teams to require deliberate permanent parent vessel occlusion, construction of a surgical bypass, or other flow-redirecting treatments that do not directly clip the aneurysm will not be excluded; these non-ISAT aneurysms are expected to be more difficult to manage with either surgical or endovascular methods.

### Inclusion/exclusion criteria

#### Inclusion criteria

Patients at least 18 years of age; at least one documented, intradural, intracranial aneurysm, ruptured within last 30 days; SAH World Federation of Neurological Surgeons (WFNS) grade 4 or less; the patient and aneurysm are considered appropriate for either surgical or endovascular treatment by the treating team.

#### Exclusion criteria

Patients with absolute contraindications of administration of contrast material (any type); patients with arteriovenous malformation-associated aneurysms; aneurysm located at basilar apex.

ISAT II will be a pragmatic trial; inclusion criteria will be kept loose and exclusion criteria minimal. The intent of ISAT II is not to repeat the initial ISAT. We expect ISAT II patients to be those for whom patient or aneurysm-related characteristics would not have led to their inclusion in ISAT, but for whom endovascular treatment is now contemplated. In other words, we are aiming to enroll patients to whom the global results of ISAT may not directly apply. As a general rule, patients previously referred to clipping despite the results of ISAT should now be considered for ISAT II. Similarly, patients considered for coiling, but with the assistance of devices that were not used in the original ISAT trial, such as stents, should be considered for ISAT II.

One exception in our pragmatic policy of as few exclusion criteria as possible is that we have chosen to exclude basilar apex aneurysms from the ISAT II study. Especially in the current climate, where surgical expertise with ruptured aneurysms in this notoriously challenging location is disappearing, this means the majority of ruptured aneurysms in this location will be treated with endovascular methods.

### Method of allocation

After confirmation of inclusion and exclusion criteria, patients will be randomly allocated 1:1 to either surgical or endovascular management, using a centralized minimization procedure. The following factors will be balanced between groups: i) age ≥70; ii) WFNS grade >3; iii) aneurysm size ≥10 mm (using maximum aneurysm diameter on cross-sectional imaging, with the outer diameter used for partially thrombosed aneurysms); and iv) posterior circulation location. The entire randomization procedure will be performed using a web-based platform available 24 hours a day.

### Justification of minimization criteria

#### Patient age

Subgroup analyses from ISAT point to a potential benefit from surgery in patients older than 70, who overall do worse than younger patients, whether they are treated one way or another. To ensure that this risk factor remains balanced between treatment groups, we have included age ≥70 (date of randomization on or beyond 70^th^ birthdate) as a minimization criterion.

#### SAH grade

Patients with Grade V aneurysm rupture, with a high mortality rate, will be excluded. Initial SAH grade is predictive of outcome [18-21]. Thus we will balance the number of poor (WFNS IV) grade SAH patients in each group.

#### Aneurysm size

Aneurysms 10 mm or larger may have greater treatment-associated risks, as well as a greater risk of incomplete occlusion and/or recurrence [18,22-25].

#### Aneurysm location: middle cerebral artery bifurcation and posterior circulation

Posterior circulation aneurysms have been associated with worse prognosis for both clipping and coiling [5]. For this reason, lesions in the different circulations will be minimized to ensure balanced groups, although we expect posterior circulation aneurysms will represent a minority of patients. Middle cerebral artery (MCA) aneurysms are common, but they were under-represented in ISAT. While they are increasingly treated with coiling, the best option remains unknown. We will monitor clinical results of treatments of MCA aneurysms separately, but this location will not be a minimization criterion.

### Anticipated use of adjunct devices

These devices are likely to be used in more difficult lesions; they necessitate the addition of a double antiplatelet regimen that may increase risks when aneurysms are treated in the early phase of SAH [26-28]. Results in this group will be monitored separately, but anticipated use of an adjunct device will not be a minimization criterion.

### Number of patients

With target alpha 0.05 and power 90%, a sample size of 1,724 patients (862 per group, no losses) would be sufficient to demonstrate a significant difference, using estimated 23% and 30% poor clinical outcome rates for endovascular treatment (EVT) and surgical management at one year, respectively (two-sided Fisher’s exact test). We expect very few losses to follow-up (<10%), and the target sample size is 1,896 patients.

### Planned patient follow-up

All patients will be seen in clinic at six to eight weeks, as part of routine follow-up care, followed by a telephone call at six months and another routine clinic visit at one year. These intervals will serve to determine mRS scores, and to inquire about possible aneurysm re-rupture. All patients will have non-invasive imaging (CTA or MRA) at six months to one year post-treatment to determine the presence of a major, saccular aneurysm recurrence. This follow-up is considered to be standard of care. There will be a study phone interview at five years to optimize long-term follow-up (Table 1).

**Table 1:** Schedule of evaluations

**Evaluation**	**Screening**	**Pre-entry**	**Entry**	**Treatment**	**Discharge**	**6 months**	**1 year**	**5 years**
Documentation of RIA	X							
Imaging*	X						X	
Informed consent			X					
Medical history		X						
Clinical assessment		X			X		X	
Neurological exam		X			X		X	
Failure to occlude aneurysm				X				
Number of days in hospital					X			
Discharge disposition					X			
Major (saccular) aneurysm recurrence							X	
Hemorrhage during FU						X	X	X
Telephone interview						X		X

*Including catheter angiography or non-invasive imaging.

### Planned analyses

The main statistical tests will involve comparisons between the probability of reaching the primary end-point (dependent survival or death at one year) with a surgical or endovascular management strategy. Descriptive statistics will be done on demographic variables and potential risk factors to compare the two groups at baseline. Means, standard deviations, and ranges will be presented for quantitative variables such as size of aneurysms and frequency tables for categorical variables (such as the number of patients with multiple aneurysms). Statistics will be broken down by center and by treatment arm. Comparison of the groups will be assessed through independent ANOVA (quantitative data) or Mantel-Haentzel and *χ*^2^ tests (categorical data). Assuming comparability of groups across centers, the primary outcome will be compared between groups using a Fisher’s exact test at one year.

Secondary outcomes will be compared using independent *t*-tests for quantitative variables and *χ*^2^ tests for categorical variables. The analyses of follow-up data will control for baseline data using logistic regression, ANCOVA or Cox regression multivariate methods. All tests will be interpreted with a 0.05 level of confidence.

### Stratification of results

Clinical outcomes will be stratified according to the minimization criteria. The Data and Safety Monitoring Board (DSMB) will regularly verify that subgroup treatment outcomes are within the confidence intervals of the study hypotheses.

### Ethics approval

The Health Research Ethics Board of the University of Alberta approved the protocol on October 1^st^, 2012 (Study ID: Pro00032613), the Institutional Review Board of the Centre Hospitalier de l’Université de Montréal approved the protocol on October 22^nd^, 2012 (Study ID: 12.136). Secondary approval will be sought from all local ethics committees. Based on the Declaration of Helsinki, written informed consent will be obtained from each participating patient or appropriate surrogate in oral and written form prior to enrollment. The ISAT II study is registered with ClinicalTrials.gov: NCT01668563.

## Discussion

In many centers, the consequences of the positive results of the original ISAT study was that endovascular ruptured aneurysm treatment was given the right of first refusal for all aneurysms, and all patients. Proponents of endovascular therapy often assert that the technology (and thus outcomes) will have improved since ISAT, which was interrupted more than 10 years ago, for the benefit of all patients. Not only has this never been proven, there is reason to fear that extrapolation of ISAT results to many patients that would not have been included in the original trial could lead to clinical outcomes that may be inferior to surgery. Although we have designed ISAT II as a pragmatic trial, the intent is not to repeat ISAT. Our aim is to provide optimal care to those patients for whom clipping may still be the best option, although this is once more only a hypothesis that must be trialed against the new generalization that coiling, in the majority of patients, provides the best results.

Although pre-randomization, as has been used in the BRAT trial, is possible, we have chosen a classical design, with randomization only after clinicians have considered the patient eligible for both treatment options, explained both alternatives, and obtained inform consent [8]. The issue of ‘equipoise’ that could, if too narrowly understood, excessively limit recruitment is acknowledged, but other designs such as Zelen’s [29] raise additional ethical issues that we preferred to avoid.

### Questions that remain unanswered after ISAT

Patients for whom major uncertainty persists can be defined by using three overlapping categories: i) patients that were excluded from the original ISAT study, for lack of ‘equipoise’ at the time; ii) patients for whom there is an a priori clinical reason to suspect results could be different from overall trial outcomes, but for whom ISAT subgroup analyses could not provide sufficient power to support a definitive conclusion; iii) patients that can now be treated using an endovascular approach as a result of the availability of new devices that were not available at the time of ISAT.

### Patients excluded from ISAT

More than 9,559 aneurysms were screened, but only 2,143 patients were enrolled in the original ISAT study [5]. The applicability of the study results to the type of patients that were screened but excluded remains in doubt. Subgroup analyses show that a variety of aneurysms and clinical situations were under-represented in the original ISAT study [30]. MCA bifurcation aneurysms are readily accessible by surgery, while their anatomy may sometimes be less favorable for simple coiling, at least during the years that ISAT was conducted. MCA aneurysms were likely excluded because they were preferentially clipped. Due perhaps to perceived difficulties with surgical access, an insufficient number of posterior circulation aneurysm (only 2.7% of the total number) were included in ISAT. Posterior circulation aneurysms, and especially basilar bifurcation aneurysms, were likely preferentially coiled [31]. As a result, a substantial number of clinicians remain unsure about the best management of MCA and of non-basilar, posterior circulation aneurysms.

### Patients for whom subgroup analyses remain unconvincing

Subgroup analyses are notoriously potentially misleading [32], however, they can be used to help formulate hypotheses for a new trial, especially when there are a priori reasons to suspect that results in certain subgroups may differ from the overall results.

#### Patient age

Subgroup analyses from ISAT point to an interaction with age (*P* = 0.04), with a potential benefit from surgery in patients older than 70 (RR: 1.15 (0.82, 1.61)). In younger patients, surgeons may be tempted to clip a ruptured aneurysm because the benefits of coiling may be smaller (RR: 0.91 (0.59, 1.39), while clipping may promise a lower risk of recurrence [33]. Younger patients (<40 years of age) have greater remaining life expectancy during which their risk of developing an aneurysm recurrence may be greater, while older patients (>70 years old) may have heightened treatment-related risks with either modality, with increased surgical risks, as well as more difficult and risky endovascular access.

#### Location

Subgroup analyses of ISAT showed an interaction with location (*P* = 0.01). Only 14.1% of aneurysms in the original ISAT were located on the MCA, likely because lesions in this location were preferentially treated surgically. Despite this selection, the subgroup analysis showed similar results for coiling and clipping (RR: 1.01 (0.71, 1.45)). Today, many more MCA aneurysms are coiled, often with adjunctive balloons and stents. Are these patients really better served with endovascular rather than surgical treatment?

#### Aneurysm size

Subgroup analyses of ISAT failed to show an interaction with aneurysm size (*P* = 0.4), and results for aneurysms larger than 10 mm were similar (RR: 0.96 (0.65, 1.42)). The rate of aneurysm recurrence increases as aneurysm size increases [9], and many clinicians are tempted to use stents or to treat large aneurysms with clipping, in spite of the ISAT overall results.

### Patients now treated via an endovascular strategy with new devices

Proponents of endovascular treatment sometimes justify expanding the indications of the endovascular approach on the basis of improved catheter and coil technology, although this presumption has never been reliably demonstrated. The increasing use of stents and, more recently flow-diverters, which were not tested in ISAT, may increase endovascular treatment risks; they require the additional use of a dual anti-platelet agent regimen to prevent subacute thrombotic complications, which can in turn increase risks of re-bleeding and hemorrhagic complications of ventricular drainage [26,34,35]. These devices may expand indications of EVT to wide-neck aneurysms that would not have been included in ISAT. The wider spectrum of patients and aneurysms now considered for EVT may not all experience the benefit seen in the original ISAT trial [6]. The relatively small ARR of 7.4% favoring coiling over clipping may no longer hold when the potential risks due to stents are considered.

Other considerations often influence treatment choices. Certain patients may carry a higher risk with endovascular management: very small (<4 mm) aneurysms [35,36] or wide-neck (>4 mm) aneurysms [26] (or any lesion whose treatment may require a stent) have been shown, in case series, to carry increased risks [26]. For these aneurysms, the relatively small advantage demonstrated in ISAT may not apply to current patients facing the dilemma of trying to choose the best treatment modality. On the other hand, some clinical situations may be more favorable to surgical management. This includes patients presenting with a large but non-life threatening intraparenchymal hematoma, or those with multiple aneurysms accessible through one craniotomy. Many questions regarding when coiling or clipping is appropriate remain, and a number of patients are proposed surgery, despite ISAT, and all without convincing evidence. On the other hand many patients are proposed EVT, using devices that have never shown superiority to clipping. Clinicians are presently forced to make case-by-case decisions based on unreliable estimates of the respective risks and benefits of each intervention. A more prudent, systematic approach is in order. The essence of scientific medicine is to question the hypotheses behind our as-of-yet not validated actions. We believe we need another pragmatic trial, which is the best way to offer the best possible care in the presence of uncertainty [37].

### When should trials be interrupted?

If so much uncertainty remains after ISAT, one may ask, at least in retrospect, why was this successful, international collaboration interrupted? Why did the SAH patient randomization process, finally under way in ISAT, not simply continue? The difficult-to-initiate process of randomizing SAH patients could easily have continued in participating centers, with a more refined question, immediately, for patients for whom the uncertainty persisted. The false notion that trials (presented here as the ethically most acceptable means to deal with patient management uncertainties), must be interrupted as soon as a convincing result is shown for the primary hypothesis, is hopefully a soon-to-be archaic notion that clinical research is foreign to medical care. The problem is that once a trial like ISAT is interrupted, the community of recruiting clinicians goes into hibernation, often for many years, until the momentum is gathered anew to launch another trial to address those questions which emerge as soon as the initial trial results are published. During this quiescent period, non-validated practices and dogmas quickly take over the field, rendering the process of trial initiation fearsomely difficult. We must remember that clinical sciences differ from ‘pure’ sciences, like physics: there are no universal laws; patient outcomes are what matters, and care must constantly be reappraised. The verdict of even a landmark trial does not close the book on the subject matter. On the contrary, it opens the door to multiple, more specific trial-able questions. With this modern vision, properly designed trials would provide optimal care in the presence of uncertainty, and the role of the DSMBs would be modified. Instead of suggesting trial termination, they would suggest interruption only for that one particular question, while pointing out where the line of questioning of the ongoing trial must continue. The current organization of clinical research, which centers on singular questions addressed one by one, should be replaced with a broader enterprise embracing programs of a wider scope and aim. Once a network of centers is well-organized and delivers reliable answers to pertinent clinical questions, it should pursue a more general goal: to provide transparent, optimal clinical care, proposing randomized studies to all patients for whom the results of an up-to-date randomized trial are not available.

### A single large trial or multiple small trials

One of the difficulties specific to our field is the relatively small number, and heterogeneity of patients with rup-tured intracranial aneurysms, which renders well-powered randomized studies addressing specific questions difficult to complete. To mitigate this difficulty, in the spirit of the original ISAT study, we have chosen to keep inclusion criteria broad. This choice of study design has two implications: the first is that at best, a general answer to the question will be obtained, which can, at some future time, be refined by other studies with more narrow inclusion criteria. Although the overall number of patients for whom the uncertainty persists is quite large (perhaps in the range of 40% or more), the reasons why these patients are not typical original ISAT cases are multiple and various. For some clinicians, each individual reason (ie: giant aneurysms, very small aneurysms) could be considered as distinct research questions, and that ISAT-II combines heterogeneous groups that they judge should not be lumped into an overall outcome result, for fear of averaging results that would normally pull in opposite directions. There may be no universally acceptable answer to this problem. Others may fear that by the sheer force of the law of large numbers differences in study population may not be clearly highlighted in demographic statistics or results and we may end up simply ‘repeating ISAT’. Except for depriving patients from a treatment that would have already been shown superior in a previous trial, a possibility that is explicitly excluded in the objectives and hypotheses of ISAT II, what this concern amounts to in actual practice remains unclear. We must, however, remember the first goal of care trials: to protect present patients from unjustified beliefs and hypotheses, and offer optimal care in the presence of uncertainty [Raymond J, Darsaut TE, Altman DG: Introducing care trials. J Clin Epidemiol 2012. Provisional acceptance]. From an organizational perspective, it is easier to propose a single inclusive large trial than to multiply small trials with narrow selection criteria. It is possible, though, to pre-specify subgroups that will separately be monitored by the DSMB. Thus, the second implication of the ISAT II design choice is that the DSMB must regularly be provided with subgroup-specific safety data to ensure that an emerging obvious discrepancy in clinical outcome will be addressed in a timely manner in order to prevent additional patient morbidity. A randomized context is the only way to firmly establish that one particular treatment modality ought not to continue to be offered to a particular subgroup of patients.

### The preservation of surgical expertise to offer optimal treatments to all patients

Since publication of ISAT results, neurovascular surgeons have increasingly been trained in endovascular techniques [38]. As patients treated with surgical clipping decreases, the open surgical expertise may become difficult to acquire, and there is a real danger that it may disappear altogether, as has virtually happened in some areas in Europe. So-called minimally-invasive treatments are not always safer, as has been recently borne out with carotid stenting [39]. While hundreds of thousands of patients were treated with this ‘minimally-invasive’ option, randomized trials comparing carotid stenting and endarterectomy have repeatedly demonstrated a higher incidence of stroke with stenting. In the same vein, there is a risk that ruptured aneurysm patients today may be treated with endovascular techniques while clipping offers a safer, more effective alternative. While EVT continues to be touted as the way ‘of the future’, there is a real chance that it may not offer the best treatment today. We must be careful to distinguish real progress, with better patient outcomes, from self-fulfilling prophecies; when neurosurgical aneurysm clipping is no longer taught to trainees and high quality clipping skills are no longer available, endovascular aneurysm treatments will become the best treatment, but by default rather than properly demonstrated merit. We owe it to patients to continue the long and often difficult process of training the next generation of neurovascular surgeons to practice scientific medicine and to ensure that we abandon treatment modalities only once alternatives have been proved better in almost all if not all circumstances.

### Trial status

ISAT II is currently recruiting patients at several Canadian centers, and the protocol is under ethics board review at other international sites.

## Abbreviations

ANCOVA: Analysis of covariance; ANOVA: Analysis of variance; ARR: Absolute risk reduction; CTA: Computerized tomographic angiography; DSMB: Data and safety monitoring board; EVT: Endovascular treatment; ISAT: International Subarachnoid Aneurysm Trial; MCA: Middle cerebral artery; MRA: Magnetic resonance angiography; mRS: Modified ranking scale; RR: Relative risk; SAH: Subarachnoid hemorrhage; WFNS: World Federation of Neurological Surgeons.

## Competing interests

The authors (TD, AJ, RK, JR) declare that they have no competing interests. RK was PI on the original ISAT study.

## Authors’ contributions

TD, AJ, and JR conceived of and designed the trial. RK consulted and advised on trial design. TD and JR drafted the manuscript. All authors read and approved the final manuscript.

## Financial disclosure

TD and JR are the principal investigators of the ‘Canadian UnRuptured aneurysm Endovascular versus Surgery trial – the CURES study’ funded by the Canadian Institute of Health Research (CIHR) (Grant MOP-119554). JR is the principal investigator of the ‘Patient prone to Recurrence after Endovascular Treatment - PRET study’ funded by Microvention Inc. (investigator-led study; unrestricted research grant).
